# Cough and Asthma

**DOI:** 10.2174/157339811794109327

**Published:** 2011-02

**Authors:** Akio Niimi

**Affiliations:** Department of Respiratory Medicine, Graduate School of Medicine, Kyoto University, Kyoto 606-8507, Japan

**Keywords:** Chronic cough, eosinophils, asthma, cough variant asthma, airway inflammation, inhaled corticosteroid.

## Abstract

Cough is the most common complaint for which patients seek medical attention. Cough variant asthma (CVA) is a form of asthma, which presents solely with cough. CVA is one of the most common causes of chronic cough. More importantly, 30 to 40% of adult patients with CVA, unless adequately treated, may progress to classic asthma. CVA shares a number of pathophysiological features with classic asthma such as atopy, airway hyper-responsiveness, eosinophilic airway inflammation and various features of airway remodeling. Inhaled corticosteroids remain the most important form of treatment of CVA as they improve cough and reduce the risk of progression to classic asthma most likely through their prevention of airway remodeling and chronic airflow obstruction.

## INTRODUCTION

Cough is a very common complaint for which patients seek medical attention [1, 2]. A number of guidelines defined chronic cough as cough lasting for 8 weeks or longer [1, 3-5]. As such, chronic cough can lead to impaired quality of life [6]. Asthma is a disease in which the airways narrow excessively in response to various stimuli in the presence of airway hyper-responsiveness (AHR) and eosinophilic airway inflammation. In “classic asthma” (CA) variable airflow obstruction typically leads to symptoms such as wheeze, dyspnea and cough. In ‘variant asthma’ originally described by Glauser in 1972 [7] and subsequently re-named, by Corrao *et al*. [8] as ‘cough variant asthma’ (CVA) cough can be the sole presenting symptom. CVA remains one of the commonest causes of chronic cough worldwide [2, 9]. More importantly, in CA cough may be associated with worse prognosis [10-12]. This review article will discuss various subtypes of asthma and associated eosinophilic airway disorders such as CVA, non-asthmatic eosinophilic bronchitis (NAEB); originally termed eosinophilic bronchitis without asthma and atopic cough [13-19].

## COUGH AND ASTHMA

Cough is a major symptom of asthma. Cough in asthma can be classified into three categories CVA, cough-predominant asthma and cough that persists despite standard therapy with inhaled corticosteroids and bronchodilators [19, 20]. CVA is a subtype of asthma that usually presents solely with cough without any other symptoms such as dyspnea or wheezing [8]. In cough-predominant asthma cough is the most predominant symptom but other symptoms are also present such as dyspnea and/or wheeze [19-21]. These symptoms can be elicited on careful clinical history and examination. The third subtype is defined as cough that persists despite the control of other symptoms such as wheeze and breathlessness with standard treatment such as inhaled corticosteroids (ICS) and beta-agonists. There are two subtypes in this category. In the first subtype cough is responsive to anti-mediator drugs such as leukotriene receptor antagonists, histamine H1 receptor antagonists and thromboxane synthesis inhibitors or receptor antagonists. The inflammatory mediators blocked by these agents are likely involved in the development of cough [22, 23]. Cough in this subtype is considered a manifestation of asthma, which is refractory to ICS and bronchodilators. The other subtype is cough due to concomitant conditions such as gastroesophageal reflux disease (GERD). Co-existence of GERD with asthma or CVA is fairly common, and cough may subside with anti-reflux medications [24]. Such phenomena may be explained by “cough-reflux self-perpetuating positive feedback cycle” leading to vicious cycle of cough and reflux [25]. As subjective measures of cough (cough scores and visual analogue scale) and cough reflex sensitivity are poor surrogates for objective cough frequency and cough-related quality of life assessment may be more appropriate when assessing cough [26].

## MUCUS HYPER-SECRETION IN ASTHMA AND CHRONIC COUGH

**Fig. (1):**
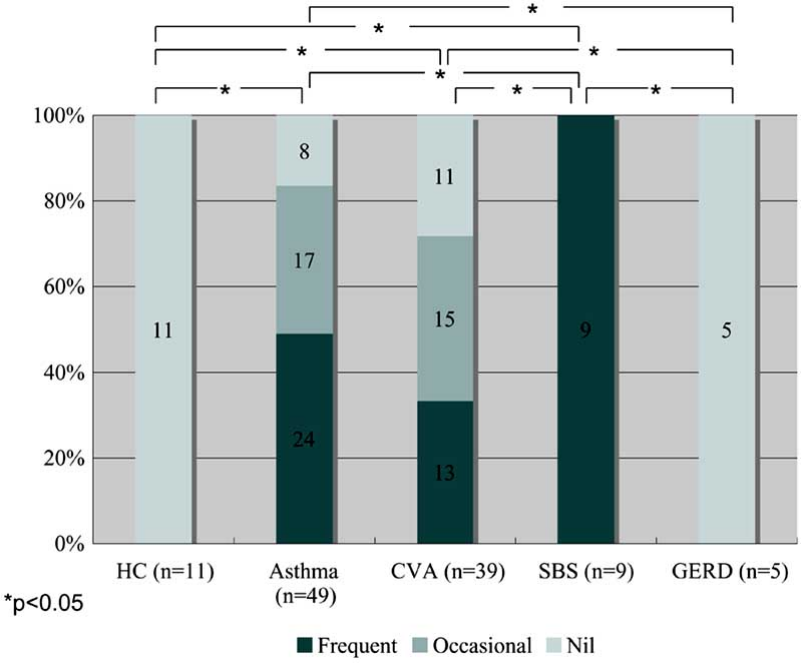
Frequency of sputum symptom in patients with asthma and chronic cough of various causes, who succeeded in sputum induction for measurement of mucin in the supernatant (ref. [32]). CVA=cough variant asthma; SBS=sinobronchial syndrome; GERD=gastroesophageal reflux disease.

**Fig. (2):**
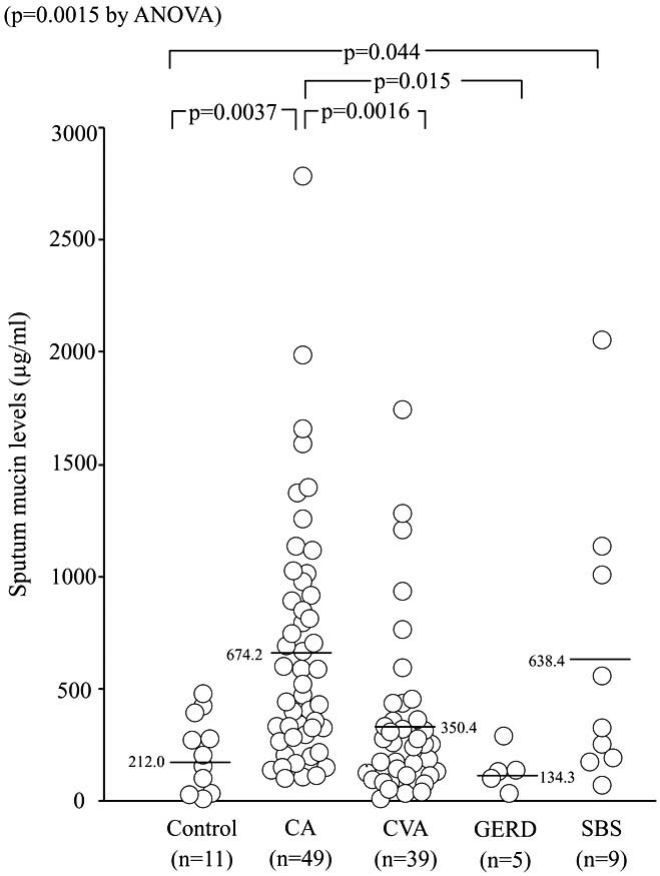
Induced sputum levels of muciun in patients with asthma and chronic cough (ref. [32]).

**Fig. (3):**
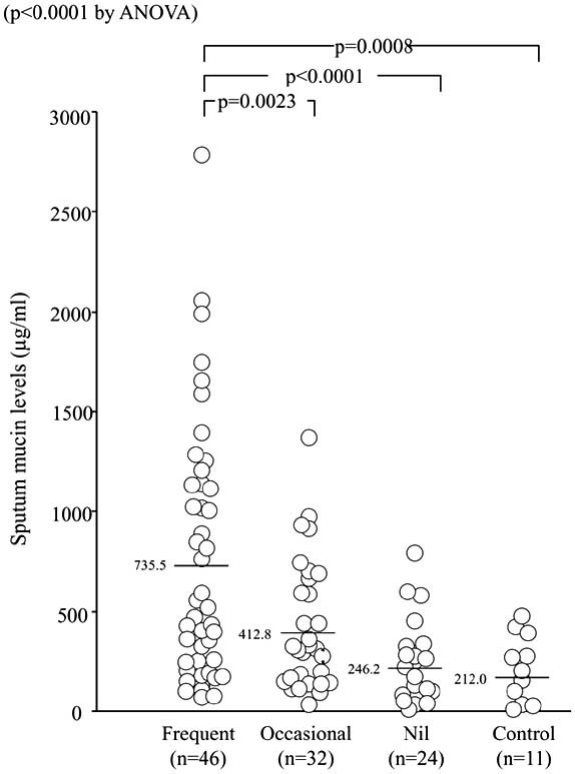
Sputum mucin levels in patients of all disease groups reclassified according to their frequency of sputum production and in controls (ref. [32]).

Cough in asthma is typically dry or minimally productive, but it may also be associated with hyper-secretion of mucus. Mucus hyper-secretion in asthma may be potentially related with steeper decline of pulmonary function [27] and fatal disease [28]. Measurement of secreted mucin in sputum has been reported in asthma [29], but not in chronic cough, which may involve goblet cell hyperplasia of bronchial epithelium with variable sputum production [30, 31]. We conducted a cross-sectional study to examine mucin levels of induced sputum supernatant in 49 patients with CA, 53 with chronic cough (39 with CVA, 9 with sinobronchial syndrome, 5 with GERD) and 11 healthy subjects [32]. Sinobronchial syndrome (SBS) was defined as chronic sinusitis complicated by neutrophilic inflammation of the lower airways [2]. An ELISA method was used [32] to detect total levels of various types of airway mucin such as MUC5AC and MUC5B [29]. Sputum symptom was semi-quantified by using a questionnaire. Sputum production was more prevalent in patients with CA, CVA, or SBS than in those with GERD and the controls. Whilst all SBS patients complained of frequent sputum production, none of the GERD patients reported sputum production, resulting in statistical differences for these two groups when compared with other disease groups (Fig. **1**) [32]. Notably, 13 of 39 CVA patients reported frequent sputum production. This may not be a true reflection of the clinical features of CVA, because only patients that succeeded with sputum induction were enrolled, possible resulting in selection bias [33]. However, it is important to notice that a subset of patients with CVA presents with productive cough. Sputum mucin levels were higher in CA and SBS than in the controls. They were also higher in CA than in CVA and GERD, but not different among the latter groups and the controls (Fig. **2**) [32]. When the four disease groups were combined, patients with frequent sputum production had greater mucin levels than those with occasional or no sputum production, or controls (Fig. **3**) [32]. These results indicated that the difference in mucin levels among subjects reflected the degree of mucus hyper-secretion. Interestingly, patients with CA showed negative correlations of mucin levels with respiratory resistance indices on impulse oscillation [34] and with airway sensitivity to methacholine [35], possibly indicating protective effects of airway-secreted mucin in asthma [32].

## COUGH VARIANT ASTHMA

In CVA cough is the sole presenting symptom. CVA is characterized by AHR. It responds to bronchodilators such as beta-agonists and theophyllines [8, 19, 20]. In Japan, CVA remains the most common cause of chronic cough followed by SBS, GERD and AC [2, 19, 20, 36-38]. The ‘health insurance for all’ in Japan allow patients to visit a specialist without prior referral from a general practitioner. The patients in fact prefer to be assessed by a specialist [2]. In the majority of patients with CVA cough can be controlled with inhaled corticosteroids. General practitioners in Japan are less likely to prescribe inhaled corticosteroids than those in Western countries [39]. This may partially explain the high prevalence of CVA in Japan.

## ATOPIC FEATURES

Seasonal variation of symptom is very common in CVA, which may implicate an involvement of atopy. We compared 74 CVA patients with 115 CA patients with wheezing with regard to total and specific IgE levels of 7 common aeroallergens [40]. The two groups of asthmatics were sensitized to one or more allergens at a similar prevalence (60% *vs* 67%). However, patients with CA had higher total IgE, larger numbers of sensitized allergens, and higher rates of sensitization to a number of allergens than did patients with CVA. The results revealed no specific antigen of CVA with higher sensitization rate than CA [40]. A literature review indicates that the prevalence of atopy in CVA, as defined by the presence of at least one positive serum specific IgE or skin test response to common aeroallergens, ranges from 40 to 80% [16, 17, 32, 40-44].

## PHYSIOLOGICAL FEATURES

Pulmonary function tests of CVA patients show normal to near normal results of peak expiratory flow (PEF) or FEV_1_, but when compared with healthy subjects or patients with post-infectious cough, these values may be slightly but significantly lower [41]. Mild diurnal change of PEF or its fluctuation in parallel with coughing may be observed [45], but to a lesser degree than that seen in CA. Reversibility of FEV_1_ with beta-agonist is smaller in CVA than in CA, because baseline FEV_1_ values are normal or nearly normal in the majority of CVA patients. In addition to these facts, CVA is the only cause of chronic cough that is responsive to bronchodilators [5, 46]. It is thus suggested that coughing of CVA may be due to bronchoconstriction, but the detailed causal relationship involved in cough and bronchoconstriction remains unknown. A recent animal experiment has suggested that cough due to bronchoconstriction is mediated *via *rapidly adapting receptors, but not C fibers [47].

AHR of CVA patients has been considered similar to or milder than that of CA patients. In adult CVA patients, airway sensitivity (threshold dose of methacholine to increase respiratory resistance) and airway reactivity (slope of methacholine - respiratory resistance dose response curve) of a tidal breathing method [35] were both smaller than in CA patients [48]. In children, only airway reactivity was smaller in CVA patients than in CA patients [49], possibly explaining the absence of wheezing in CVA. As a whole, the physiological abnormalities of CVA are more modest than those of CA. However, this does not mean that CVA is a milder form of asthma, because CVA patients are often more difficult to manage than CA patients who predominately present with wheeze. Cough receptor sensitivity, most commonly assessed by inhalation of capsaicin, may or may not be heightened in CVA as compared with healthy controls, and may decrease with treatment with leukotriene receptor antagonists [50] while remain unchanged with ICS treatment [51]. This might be associated with excellent antitussive effect of the former class of drugs [52], but the details of its mechanism remain unknown.

## PATHOLOGICAL FEATURES

**Fig. (4):**
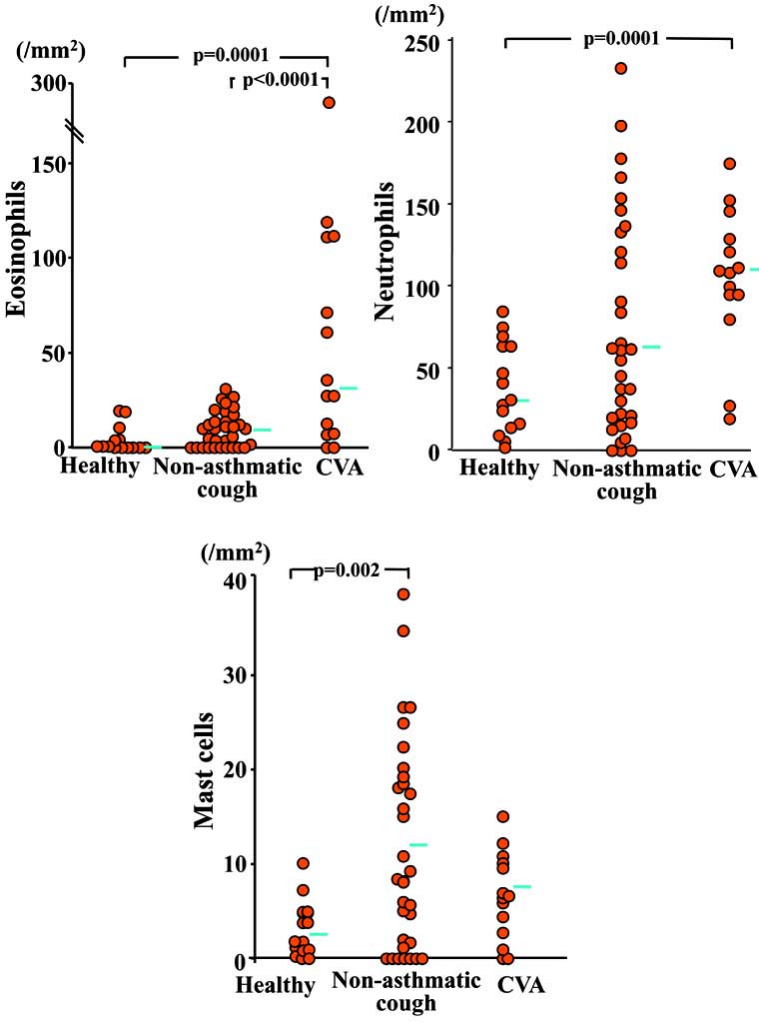
Number of eosinophils, neutrophiils and mast cells in the submucosa of bronchial biopsy specimens obtained from patients with CVA (n=14), those with non-asthmatic chronic cough (n=33; 6 with postnasal drip/rhinitis, 5 with GERD, 3 with bronchiectasis, and 19 with idiopathic disease) and 15 healthy subjects [30].

In patients with CVA, eosinophils are increased in the sputum [51, 53], bronchoalveolar lavage (BAL) fluid and bronchial mucosal tissue [41]. The magnitude of this increase correlates with the severity of disease as defined by symptom and treatment required to achieve control [41]. For biopsy specimens of central airway mucosa and BAL fluid recovered from peripheral airways and lung parenchyma, the degree of eosinophilia is similar between CA and CVA, indicating a similarity in the site of inflammation [41]. One study showed an increase of neutrophils as well as eosinophils in the airway mucosa of CVA patients (Fig. **4**) [30], and such concomitant increase of both cells may lead to more severe disease characterized by refractoriness to treatment with ICS [54]. Mast cells, an important source of tussive as well as fibrogenic mediators, was increased in the airway mucosa of non-asthmatic chronic cough patients but not CVA patients (Fig. **4**) [30].

**Fig. (5):**
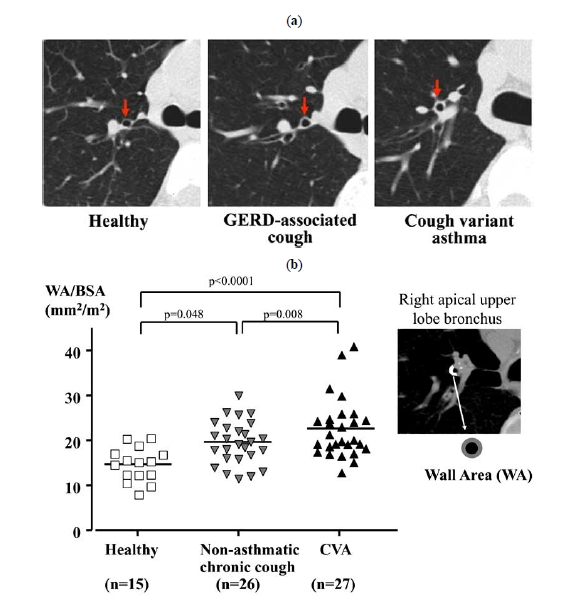
Airway wall thickening in patients with CVA (n=27) and non-asthmatic chronic cough (n=26; 8 with SBS, 3 with GERD, 3 with post-infective cough, 11 with unexplained cough, and 1 with combined SBS and GERD), as indicate by helical CT scans of right apical upper lobe bronchus (**a**). Airway wall thickness (wall area/body surface area) as quantified by automatic analysis shows that airway walls of patients are thickened as compared with healthy subjects (n=15), to a greater degree in patients with CVA (**b**) [42].

Similar to asthma, in CVA structural changes such as sub-epithelial thickening, goblet cell hyperplasia and vascular proliferation have been demonstrated on mucosal biopsies [30, 55, 56]. These changes may be secondary to airway inflammation. Pathophysiological significance of these changes has been indicated in asthma, and early anti-inflammatory treatment is also recommended in CVA. However, they may also be a consequence of long-term mechanical stimulation by coughing [30, 56, 57], because most of these changes are also present in subjects with non-asthmatic chronic cough [30, 56]. Increased inflammatory mediators (e.g. histamine, prostaglandins D_2_ and E_2_ and leukotrienes C4, D4 and E4) [23], increased expression of capsaicin receptor TRPV-1 [58], and decreased pH of airway lining fluid that may activate TRPV-1 [59] may play a role in the development of cough. One study showed a similar pattern of sputum markers (cellular and humoral) between CA and CVA [60]. A computed tomography (CT) study has revealed airway wall thickening, a feature of CA [61], in patients with CVA (Fig. **5a**, **b**) [42]. This may reflect the net effect of airway remodeling features discussed above. However, airway wall thickening is also present in patients with non-asthmatic chronic cough although to a lesser degree (Fig. **5b**) [42], which is consistent with the biopsy studies [30, 56].

## DIAGNOSIS

CVA is characterized by AHR and responsiveness to bronchodilators, but the presence of AHR is only consistent with, but not diagnostic of, CVA [46]. Improvement of cough with bronchodilators such as beta-agonists is the essential diagnostic feature of CVA, as demonstrated by the double-blind controlled study by Irwin *et al. *[46]. Based on these features, the Japanese Respiratory Society cough guideline considers responsiveness to bronchodilators as the key diagnostic feature of CVA [5]. Sputum eosinophilia suggests a diagnosis of CVA [51, 53], as well as AC or NAEB. However, only 30% of CVA patients fulfill the criteria of sputum eosinophilia as defined by >3% of leukocytes [14] in our experience (unpublished data), and its absence does not exclude the diagnosis of CVA. Exhaled NO levels may also be elevated and useful in the diagnosis of CVA [62].

## TREATMENT AND PROGNOSIS

After the diagnosis is established, treatment of CVA is essentially the same as in CA [5]. Bronchodilators (short-acting inhaled β_2_ agonists or theophyllines) may be used especially in patients with intermittent cough. However, as in CA eosinophilic airway inflammation and remodeling are present in CVA, ICS are the first line treatment in CVA especially in those patients who have persistent cough [30, 41, 55, 56]. There are no data currently available regarding the choice of ICS, its dose or duration that should be used for the treatment of CVA [3, 52, 63]. If ICS mono-therapy is insufficient, other agents can be added such as long-acting β_2_ agonists, slow-release theophylline, or leukotriene receptor antagonists [5]. Effectiveness of mono-therapy with leukotriene receptor antagonists has been reported possibly through its anti-inflammatory effects [50, 64]. Occasionally, for acute exacerbations of CVA, a short-course of oral corticosteroids may be required. A subset of patients with CVA can develop wheeze and progresses to CA. If ICS are not used in CVA, the progression rate to CA has been reported between 30 to 40% [17, 43]. Factors that may predict the development of CA include AHR [17] and exaggerated maximal airway response to methacholine, sputum eosinophilia [65], and sensitization to allergens [40]. Early ICS treatment may reduce the risk of progression to CA [17, 43]. Avoidance of relevant allergens might also be important [40]. As in CA cough in CVA often re-occurs if treatment is discontinued [43].

Annual changes of FEV_1_ have been reported to be similar among patients with CVA (-29 ml/year by average), those with AC (-21 ml/year) and healthy subjects (-28 ml/yr). Values for CVA patients ranged from approximately -90 ml to +30 ml [18]. In our 3- year follow-up [19], annual changes of FEV_1_ were -2 ± 36 ml in 7 patients with mild CVA (coughing episodes interfering with usual activities or sleep 3 times or less yearly), and -62 ± 35 ml in 4 patients with difficult-to-control disease (4 or more such episodes yearly)(p=0.014). The difficult-to-control group was treated with higher doses of ICS than the mild group, reflecting their severity [19]. Although this is a small study, these results may be consistent with recent studies in CA that showed a positive relationship between the number of asthma exacerbations and progressive loss of FEV_1_ [66].

## DISORDERS RELATED TO CVA

Atopic cough as proposed by Fujimura *et al. *[16-18, 44, 51] presents with bronchodilator-resistant dry cough associated with an atopic constitution. It is characterized by eosinophilic tracheobronchitis and cough hypersensitivity. However, there is absence of AHR and variable airflow obstruction. AC usually responds to ICS treatment. These features are shared by NAEB [13-15, 53]. However, AC lacks BAL eosinophilia [16]. Unlike CVA [17, 41, 43, 44] and NAEB [19, 67, 68], AC rarely progresses to CA with wheezing [17]. Histamine H_1_ antagonists are effective in AC [69], but their efficacy in NAEB is unknown. The involvement of airway remodeling and accelerated decline of lung funciton, which has been shown in CVA [19, 30, 42, 55] and NAEB [15, 70, 71], is unknown for AC. NAEB thus significantly overlaps with AC, but might also include milder cases of CVA with very modest AHR. The clinical and pathological features of eosinophilic airway disorders including CA are summarized in Table **1**. The confusion, or lack of consensus, in these related entities may be affecting the etiology of chronic cough reported from various countries [2, 19].

## CONCLUSIONS

CVA is one of the most common causes of chronic cough. CVA therefore should be considered in patients presenting with persistent cough. The role of inhaled corticosteroids in CVA is very important. The inhaled corticosteroids not only control cough in CVA but also they may prevent the development of wheeze, airway remodeling and chronic airflow obstruction.

## Figures and Tables

**Table 1:** Clinical and Pathological Features of Eosinophilic Airway Disorders

	Classic Asthma	CVA	NAEB	AC
Symptoms	Cough, SOB, wheeze	Cough only	Cough (often with upper airway symptoms)	Cough only
Atopy*	60-80%	40-80%	20-70%	40-50%
Variable airflow limitation	+	±	—	—
AHR	+	+	—	—
Cough hypersensitivity	—~↑	—~↑	↑	↑
Response to bronchodilator	+	+	unknown	—
Response to corticosteroid	+	+	+	+
Response to H_1_ antagonists	±	±	unknown	+
Rapid decline of lung function	+	±	±	—
Progression to classic asthma	NA	30%	10%	rare
Sputum eos↑(>3%)	usually	usually	always (by definition)	usually
Exhaled NO	↑	↑	↑	→
Submucosal eos	↑	↑	↑	↑
BAL eos	↑	↑	↑	→
Mast cells in ASM	↑	→	→	unknown
Subepithelial thickening	+	+	+	unknown
Vascular proliferation	+	+	+	unknown

* Defined by the presence of at least one positive serum specific IgE or skin test response to common aeroallergens.
